# Sex Differences in Rhesus Monkeys’ Digit Ratio (2D:4D Ratio) and Its Association With Maternal Social Dominance Rank

**DOI:** 10.3389/fnbeh.2018.00213

**Published:** 2018-09-21

**Authors:** Alexander Baxter, Elizabeth K. Wood, Parker Jarman, Ashley N. Cameron, John P. Capitanio, J. Dee Higley

**Affiliations:** ^1^Department of Psychology, Brigham Young University, Provo, UT, United States; ^2^California National Primate Research Center (CNPRC), Davis, CA, United States; ^3^Department of Psychology, University of California, Davis, Davis, CA, United States

**Keywords:** 2D:4D digit ratio, sex differences, prenatal androgen exposure, rhesus monkey, social dominance rank, maternal androgens, organizational effects

## Abstract

Prenatal androgen exposure (PAE) plays a pivotal role in masculinizing the developing body and brain, and extreme exposure may contribute to autism, anxiety disorder and schizophrenia. One commonly used biomarker for PAE is the pointer-to-ring-finger digit length (2D:4D) ratio. Although this biomarker is widely used in human studies, relatively few studies have investigated 2D:4D ratio in nonhuman primates, particularly rhesus macaques (*Macaca mulatta*), one of the most commonly used animals in biomedical research. Thus far, data suggest that sexual dimorphism in 2D:4D ratio may be in the opposite direction in some monkey species, when compared to the pattern exhibited by humans and great apes. Using a large sample size, we investigated whether rhesus monkeys’ 2D:4D ratio shows the same sex-differentiated pattern present in other Old World monkey species. We also investigated whether individual differences in 2D:4D ratio are associated with the social dominance rank of subjects’ mothers during pregnancy, and the social dominance rank the subjects attained as adults. Subjects were 335 rhesus monkeys between 3 years and 24 years of age (*M* = 6.6). Maternal dominance rank during pregnancy and subjects’ adult dominance rank were categorized into tertiles (high, middle and low). Results showed that, across both hands, male rhesus monkeys exhibited higher 2D:4D ratio than females, a pattern consistent with other monkey species and a reversal from the pattern typically observed in humans and apes. This sex difference was modulated by maternal dominance rank, with female offspring of high-ranking and middle-ranking mothers exhibiting masculinized 2D:4D ratio, indicating that maternal dominance rank during pregnancy may influence levels of PAE. There was no association between subjects’ 2D:4D ratio and the social dominance rank they attained as adults. These findings show a consistent sex difference in Old World monkeys’ 2D:4D ratio that diverges from the pattern observed in apes and humans, and suggest maternal social dominance rank modulates PAE in rhesus monkeys.

## Introduction

Prenatal androgen exposure (PAE) plays a critical organizational role in masculinizing the developing body and brain. For example, during gestation, male fetus’ testes flood the womb with androgens, masculinizing the developing organism (Hughes, 2001). Beyond masculinizing genitalia, prenatal androgens shape the developing brain by binding to androgen receptors (Lombardo et al., 2012; McCarthy, 2012), thereby initiating intracellular processes that regulate gene expression (MacLusky and Naftolin, 1981; Rubinow and Schmidt, 1996) and apopotosis (see Morris et al., 2004). Consequently, variation in PAE can lead to lasting sex differences in brain organization and functioning (Lombardo et al., 2012), and can have a profound influence on post-natal behavior, personality and health (see Manning, 2011), contributing to gross sex differences, as well as within-sex variation in these traits (McCarthy et al., 2012). Studies suggest that in humans, PAE plays a role in the etiology of a number of disorders and psychiatric conditions (Manning, 2011). For example, in males, high levels of PAE are implicated in the occurrence and severity of autism spectrum disorder (Manning et al., 2001) and low levels of PAE are associated with increased anxiety (De et al., 2006; Evardone and Alexander, 2009). In both sexes, low levels of PAE may be associated with schizophrenia (Collinson et al., 2010; Paipa et al., 2018) and high levels of PAE may be associated with attention deficit hyperactivity disorder (Stevenson et al., 2007; Martel et al., 2008). While PAE is only one of the factors contributing to these disorders, and the activating effects of postnatal androgens may also contribute (Arnold and Breedlove, 1985), a better understanding of the structural and functional effects of PAE on the brain could potentially lead to better preventions, treatments and interventions, as well as a better understanding of sex differences in the brain and in behavior.

While testes are the primary source of PAE for male fetuses, female fetuses are also exposed to androgens (Herman et al., 2000), likely as a byproduct of the fetus producing other steroid hormones and from circulating maternal androgens that cross the placenta (see Christine Knickmeyer and Baron-Cohen, 2006), although levels of exposure are typically much lower for females than for males, and, in most cases, do not result in the same degree of masculinization as that typically seen in males (Hughes, 2001). Thus, for male and female fetuses, individual variation in levels of PAE may result from genetic differences in steroid hormone production (Warrington et al., 2018), as well as factors that affect maternal androgen levels, such as maternal age (Kallak et al., 2017), maternal stress levels (Dahlöf et al., 1978), or disorders that dysregulate maternal androgen levels, including congenital adrenal hyperplasia (Speiser and White, 2003) and polycystic ovary syndrome (Franks, 1995). Patterns of maternal behavior and personality may also affect levels of PAE, as highly competitive and socially-dominant behavior is associated with higher levels of circulating androgens (for review see Mazur and Booth, 1998), although it is unknown whether maternal personality has direct effects on PAE. Variation in the fetus’ androgen receptor gene may also mediate the relative sensitivity of the fetus to levels of PAE (Manning et al., 2003; Berenbaum et al., 2009; Warrington et al., 2018).

One common proxy measure for levels of PAE is the second-to-fourth-finger length (2D:4D) ratio (see Manning, 2011 for a comprehensive review). In many species, PAE induces growth in the fourth finger (ring finger) without inducing growth in the second finger (index finger; see Manning, 2011; Zheng and Cohn, 2011). This is likely due to differences in the concentration of androgen receptors in these digits, with many receptors in the fourth finger and relatively few receptors in the second finger (Zheng and Cohn, 2011). Thus, as prenatal androgens masculinize the typical male fetus, they bind to androgen receptors in the fourth finger and induce growth, likely by altering the expression of genes related to bone growth (see Manning et al., 2003; Zheng and Cohn, 2011; Warrington et al., 2018). For this reason, human males’ ring fingers tend to be longer than their pointer fingers, resulting in a low 2D:4D ratio (<1.0), while females’ ring fingers tend to be shorter than or equal to their pointer finger in length, resulting in a high 2D:4D ratio (≥1.0; see Manning, 2011). Although this sex difference is seen in aggregate, and likely reflects the higher levels of PAE that males experience when compared to females (Manning, 2011), variation in PAE within each sex can lead to dose-dependent variation in 2D:4D ratio. Hence, males that exhibit high (female-typical) 2D:4D ratio likely experienced lower levels of PAE than did typical males and females that exhibit low (male-typical) 2D:4D ratio likely experienced higher levels of PAE than did typical females.

There is much evidence for the reliability and validity of 2D:4D ratio as a biomarker for PAE. Importantly, in humans, adult-like sex differences in 2D:4D ratio emerge prenatally (Galis et al., 2010), and these sex differences have been consistently observed in infants (Knickmeyer et al., 2011; Ventura et al., 2013; Wong and Hines, 2016). Although 2D:4D ratio tends to increase slightly as children age, suggesting some postnatal effects, individual differences in 2D:4D ratio remain stable across development (Trivers et al., 2006; Wong and Hines, 2016). Despite slight differences in how fingers are measured across investigations, a study providing researchers that study 2D:4D ratio with scans of subjects’ hands found that intra-class correlations ranged from 0.7 to 0.8 (Voracek et al., 2007), indicating that while measurement error may explain why some 2D:4D ratio studies report null findings, results are likely comparable across studies. It is important to note that some have challenged the use of 2D:4D ratio as a biomarker for PAE, and argue that other postnatal or genetic factors may affect 2D:4D ratio (see Hampson and Sankar, 2012), and that sex differences in 2D:4D ratio are not large enough for it to be used as a proxy for precise levels of PAE (see Berenbaum et al., 2009). However, studies have demonstrated that in mice (Zheng and Cohn, 2011) and monkeys (Abbott et al., 2012), experimentally increasing levels of PAE leads to masculinized 2D:4D ratio. Moreover, in the monkey study, the degree to which 2D:4D ratio was masculinized correlated strongly with anogenital distance (Abbott et al., 2012), a well-established biomarker for PAE (Thankamony et al., 2016). Hence, while 2D:4D ratio is not a perfect proxy for PAE, it is a reasonable estimate and is easier to collect than direct measurements of prenatal androgen levels from amniotic fluid, which can be inconvenient and unnecessarily invasive for pregnant mothers. Moreover, a single intrauterine measurement during pregnancy may not represent the day-to-day androgen levels to which a fetus is exposed, whereas 2D:4D ratio likely represents the pooled effect of continuously high or low levels of PAE throughout gestation, and is thus more resistant to the daily variations that would decrease the accuracy of a single, direct measurement (see Breedlove, 2010).

While 2D:4D ratio has been researched extensively in humans, the relatively few studies of 2D:4D ratio in nonhuman primates have yielded mixed findings, particularly in relation to sexual dimorphism in 2D:4D ratio. Studies suggest that sexually-dimorphic patterns of 2D:4D ratio vary across primate phylogeny (Nelson and Shultz, 2010), and, for Old World monkeys, the sexually-dimorphic pattern may be reversed when compared to humans and great apes. In a study of bonobos (*Pan paniscus*) and chimpanzees (*Pan troglodytes*), McIntyre et al. (2009) found that these ape species have a human-like pattern of sex differences in 2D:4D ratio, with males exhibiting lower 2D:4D ratio than females, which suggests that the sex-differentiated pattern of 2D:4D ratio is phylogenetically-conserved across hominids. However, some studies of certain Old World monkey species suggest that the pattern of sex differences in 2D:4D ratio may be reversed when compared to humans and apes (in rhesus macaques (*Macaca mulatta*): Abbott et al., 2012; in Guinea baboons (*Papio papio*): Roney et al., 2004). For example, Roney et al. (2004) found that male Guinea baboons exhibited higher 2D:4D ratio than females. Evidence, however, is mixed, as a study of a different baboon species (*Papio hamadryas*) failed to find a sex difference in 2D:4D ratio (Huber et al., 2017). Rhesus monkeys may also show a reversed pattern of sexual dimorphism in 2D:4D ratio. As discussed above, in one study, administering prenatal androgens increased female rhesus monkeys’ right-hand 2D:4D ratio (Abbott et al., 2012), suggesting that high 2D:4D ratio indicates high levels of PAE in this species. It should be noted that this study, as well as another study of untreated free-ranging rhesus monkeys, found no naturally-occurring sex differences in 2D:4D ratio between untreated females and males (Nelson and Voracek, 2010; Abbott et al., 2012). However, the small sample sizes of these studies^1^ may have led to inadequate statistical power to detect sex differences in 2D:4D ratio. Although one study measured 2D:4D ratio in a large number^2^ of rhesus monkeys (Nelson and Shultz, 2010), the primary purpose of the study was to investigate phylogenetic differences in 2D:4D ratio across many different primate species, and sex differences in individual species were not reported. Thus, further investigation of 2D:4D ratio in a large sample of rhesus monkeys is merited, and may have clinical application. In humans, extremes in 2D:4D ratio (suggesting alterations in levels of PAE) have been linked with psychiatric disorders like autism spectrum disorder (Manning et al., 2001) and anxiety (De et al., 2006; Evardone and Alexander, 2009). Although rhesus monkeys are one of the most commonly used animal in biomedical research (see Harlow, 2008), and allow researchers to study these psychiatric disorders (see, for example, Kalin and Shelton, 2003; Capitanio, 2017; Parker et al., 2018) with greater control than is possible with human subjects (see Gibbs et al., 2007), relatively few studies have investigated 2D:4D ratio in this species, and none, to our knowledge, have investigated 2D:4D ratio in an animal model context. A better understanding of 2D:4D ratio in rhesus monkeys may guide researchers in understanding how PAE contributes to a wide variety of psychological disorders, and may lead to better assessments and treatments.

In humans and nonhuman primates, research on 2D:4D ratio suggests that the organizational effects of PAE may influence future social status and its related behavior (in humans: Manning and Fink, 2008; in rhesus monkeys: Nelson et al., 2010); however, the relationship is complex, as the mother’s socially-dominant personality and behavior may alter circulating maternal androgens, and potentially the levels of PAE to which the fetus is exposed (Nelson et al., 2012). In humans, there is a bi-directional relationship between socially-dominant behavior and circulating androgens, as high levels of circulating testosterone not only precede the motivation for social dominance (for review see Mazur and Booth, 1998), but also increase after achieving social dominance or winning in a competitive situation (Bernhardt et al., 1998). This relationship may also be dependent on contextual factors (in humans: Rowe et al., 2004) or other neurological systems related to impulse control (in rhesus monkeys: Higley et al., 1996; in humans: Kuepper et al., 2010). Because social dominance is a complex trait, rhesus monkeys offer a promising model for studying social dominance with greater control and precision (McCowan et al., 2016). Their complex social groups, strict dominance hierarchies (Lindburg, 1971), and aggressive tendencies (Thierry, 1985) make them well-suited for studying socially-dominant behavior and temperament. Paralleling research in humans, high social dominance is associated with high levels of circulating androgens in male rhesus monkeys living in stable social groups (Rose et al., 1971), a finding replicated in male (Sapolsky, 1983) and female baboons (Beehner et al., 2005), a species with a similar social organization to rhesus monkeys.

As a consequence of rank-related higher androgen levels, it is possible that high-ranking female monkeys expose their fetuses to higher levels of PAE during gestation (see Nelson et al., 2012), which may affect their offspring’s 2D:4D ratio (Howlett et al., 2014). However, to our knowledge, only one study has assessed the association between maternal dominance rank and offspring 2D:4D ratio. In an investigation of six infant baboons, Howlett et al. (2014) found that infants born to high-ranking mothers had lower 2D:4D ratio than infants born to low-ranking mothers, suggesting that maternal dominance rank may contribute to levels of PAE. This theory is further corroborated by research in spotted hyenas (*Crocuta crocuta*), a species with similar social structure to rhesus monkeys and baboons (see Holekamp and Smale, 1991 for review). In a seminal study, Dloniak et al. (2006) found that high-ranking females experienced an increase in androgens during their last trimester of pregnancy that low-ranking mothers did not experience. Because the only previous study to investigate maternal dominance rank and 2D:4D ratio in monkeys had a small sample size (Howlett et al., 2014), and, as these variables have not been studied in rhesus monkeys, the present study investigates the link between maternal social dominance rank and offspring 2D:4D ratio in a large sample of rhesus monkeys. Because PAE may, in turn, affect temperament and personality traits related to socially dominant behavior (in humans: Manning and Fink, 2008; in baboons: Howlett et al., 2014), another purpose of this study is to investigate the association between 2D:4D ratio and the social dominance rank that monkeys attain as adults. Because this association has only been studied thus far in female monkeys (see Nelson et al., 2010; Howlett et al., 2012), this study investigates a large sample of males and females.

The purpose of this study is two-fold. First, while there are substantial data showing sex differences in 2D:4D ratio across primate species, with great apes showing a similar sex difference to that seen in humans (in bonobos and chimpanzees: McIntyre et al., 2009), some studies suggest that sexual dimorphism in 2D:4D ratio is in the opposite direction for certain Old World monkey species (in rhesus monkeys: Abbott et al., 2012; in Guinea baboons: Roney et al., 2004). Thus, the first goal of this study is to investigate whether there are sex differences in rhesus monkeys’ 2D:4D ratio, and whether the direction of that difference is the same as or different from the pattern of sex differences seen in humans’ and apes’ 2D:4D ratio. Because PAE appears to increase 2D:4D ratio in rhesus monkeys (Abbott et al., 2012), we hypothesize that the pattern will be reversed when compared humans and apes, and that male rhesus monkeys will exhibit higher 2D:4D ratio than will females. The second purpose of this study is to investigate the association between 2D:4D ratio and maternal dominance rank, as well as the social dominance rank subjects attain as adults. Because research suggests that high-ranking mothers in stable social groups experience higher levels of testosterone than low-ranking mothers (Beehner et al., 2005; Dloniak et al., 2006), we hypothesize that within each sex, the offspring of high-ranking subjects will exhibit a more masculinized 2D:4D ratio than offspring of low-ranking monkeys.

## Materials and Methods

### Subjects

All procedures were in accordance with guidelines established in the *Guide for the Care and Use of Laboratory Animals* (National Research Council, 2010), and were performed in accordance with institutional and national guidelines. The measurements reported here were conducted during regularly scheduled health checks, the procedures for which were reviewed and approved by the University of California, Davis’s Institutional Animal Care and Use Committee. Subjects were *n* = 335 rhesus monkeys (120 males, 215 females) housed at California National Primate Research Center (CNPRC). Because data were collected as part of semiannual health examinations at CNPRC, subjects were selected opportunistically, based on the examination schedule. The average age of the subjects included in analyses was 6.6 years old (*SD* = 4.27, Range: [3, 24 years of age]), and subjects were only included if they were older than 2 years old. Most subjects (*n* = 314) lived in outdoor, half-acre corrals housing between 80 and 120 animals, and some (*n* = 21) lived in smaller, outdoor corrals (approximately 5 × 5 feet), housing up to 15 monkeys (for more details on housing conditions at CNPRC, see Kanthaswamy et al., 2010). These two groups were combined for analyses, as preliminary analyses showed no difference in 2D:4D ratio between outdoor housing conditions.

### Finger Measurements

Monkeys’ fingers were measured while they were sedated with 10 mg/kg ketamine hydrochloride (see Bentson et al., 2003). Subjects’ fingers were not measured if they had severe hand trauma, or if they were rousing from sedation.

To measure monkeys’ fingers, monkeys were laid on a table in alternating recumbent positions (see Abbott et al., 2012). Pilot assessments showed that the greatest source of measurement variability was a function of finger restraint. To avoid this, a technician used a wooden craft stick (11.43 cm long) to depress monkeys’ palms and fingers flat against the table for measurements. To maintain uniformity, the same technician depressed all subjects’ hands across cohort years. A second technician measured fingers using a digital caliper and, following guidelines set forth by Manning (2011), measured from the finger-crease most proximate to the palm to the most distal point of the finger, not including the nail. Subjects’ right- and left-hand second and fourth fingers were measured at least twice, until at least two measurements were obtained within 1.5 mm, and the average length of the finger was calculated by averaging the two closest measurements. Subjects’ 2D:4D ratios were calculated by dividing the average length of the second finger by the average length of the fourth finger, respectively for each hand.

Across the two cohort years, six different technicians measured subjects’ fingers as part of this study. All technicians were trained by the first author and demonstrated acceptable reliability with the first authors’ measurements (greater than 88% between rater agreement). Preliminary analyses showed that right- and left-hand 2D:4D ratio were correlated only weakly, and not significantly (*r* = 0.13, *p* = 0.063; see Table 1). For this reason, we performed all subsequent analyses of 2D:4D ratio with subjects’ right-hand 2D:4D ratio and left-hand 2D:4D ratio as repeated measures.

**Table 1:** Correlations between variables in analyses.

Variable	1. Left 2D:4D	2. Right 2D:4D	3. Age	4. Weight	5. Adult rank
Combined subjects					
1. Left-hand 2D:4D ratio	−				
2. Right-hand 2D:4D ratio	0.13	−			
3. Age	0.05	−0.07	−		
4. Weight	0.09	0.02	***0.59***	−	
5. Adult rank tertile^a^	0.01	−0.03	**0.19**	**0.28**	−
6. Maternal rank tertile^a^	−0.07	−0.04	0.11	0.03	**0.50**
Females					
1. Left-hand 2D:4D ratio	−				
2. Right-hand 2D:4D ratio	0.11	−			
3. Age	0.11	−0.003	−		
4. Weight	0.10	0.04	**0.60**	−	
5. Adult rank tertile^a^	−0.003	−0.04	−0.02	0.03	−
6. Maternal rank tertile^a^	0.02	0.01	−0.01	−0.02	**0.63**
Males					
1. Left-hand 2D:4D ratio	−				
2. Right-hand 2D:4D ratio	0.14	−			
3. Age	−0.05	−0.18	−		
4. Weight	0.01	−0.06	**0.75**	−	
5. Adult Rank Tertile^a^	−0.01	−0.12	**0.72**	**0.69**	−
6. Maternal Rank Tertile^a^	−0.14	−0.11	**0.26**	0.19	**0.30**

*The table shows the correlations between variables used in analyses. Bold indicates the correlation is significant (*p* < 0.05). The ^a^indicates Spearman Rho rank correlation was used; all other correlations are Pearson correlation coefficients. For ease of interpretation and explanation, subjects’ adult social dominance rank tertile and maternal social dominance rank tertile were reverse coded as high-ranking subjects = 3, middle-ranking subjects = 2 and low-ranking subjects = 1*.

### Determination of Social Dominance Rank

The social dominance rankings of subjects’ mothers were determined from observations conducted during the 6 months before the mother gave birth. The social dominance ranks that subjects attained as adults were determined from ongoing monthly observations conducted by trained behavioral management staff. All observers were trained by senior behavioral management staff and demonstrated reliable behavior scoring (greater than 85% agreement). The staff conducted observations by throwing several handfuls of sunflower seeds into the corral to lure the animals to the front. While the monkeys foraged, trained observers monitored the group for aggressive and submissive interactions as the monkeys competed for the limited resources, recording wins and losses. Monkeys were ranked higher if they threatened, displaced, chased, or attacked another monkey; monkeys were ranked lower if they surrendered resources or their location, or showed submissive behaviors like lip-smacking, fear grimacing, or moving out of proximity of another monkey. Subjects were ranked separately by sex, with the alpha male and alpha female ranked as 1. Because young monkeys often share the dominance rank of their mother before puberty (Bernstein and Williams, 1983), the observers did not rank subjects younger than 1 years old. Social dominance rank data were not collected for animals housed in the smaller outdoor corrals.

Because some social groups were larger than others, social dominance ranks were categorized into tertiles. Tertiles were determined based on the cumulative percentile at which each subjects’ dominance rank fell within their social group and sex. Subjects whose dominance rank fell at or below the 33rd percentile were considered high-ranking. Subjects whose dominance rank fell between the 33rd percentile and the 66th percentile were considered middle-ranking. Subjects whose dominance rank fell above the 66th percentile were considered low-ranking.

### Control Variables

For analyses with 2D:4D ratio as the dependent variable, subjects’ age, weight, and social group were statistically controlled. Although age and weight were not directly correlated with left-hand 2D:4D ratio or right-hand 2D:4D ratio (see Table 1 for a summary of correlations between variables), as was previously done in studies of social dominance rank and 2D:4D ratio in rhesus monkeys, all analyses with significant effects were repeated with age and weight as covariates (see Nelson et al., 2010). This was also done because some correlations were significant between control variables, maternal social dominance rank, and subjects’ adult social dominance rank (see Table 1). Because preliminary analyses revealed a significant interaction between social group (field cage in which the subjects were living at the time of measurement) and hand (*F*_(5,198)_ = 43.73, *p* = 0.003), such that social-group differences in 2D:4D ratio were present in the right-hand 2D:4D ratio (*F*_(6,281)_ = 4.03, *p* = 0.001), but not in the left-hand 2D:4D ratio (*F*_(6,253)_ = 1.14, *p* = 0.34), all analyses were repeated using subjects’ social group as a random effect to control for possible genetic or social group-differences that could influence 2D:4D ratio. However, since neither social group, age, nor weight contributed to any of the models, we do not report analyses with these variables.

### Tests of Independence Between Social Dominance Rank and Maternal Dominance Rank

Because preliminary analyses showed that subjects’ adult social dominance rank was correlated with their mother’s social dominance rank at birth (see Table 1), Pearson Chi Squared Tests of Independence were performed to assess whether these two variables were independent of each other. This analysis was performed separately for each sex because in free-ranging populations patterns of inheriting maternal dominance rank differ between males and females (Lindburg, 1971), a pattern also evident in our data by the stronger correlation between subjects’ adult dominance rank and the dominance rank their mother held at birth in females compared to males (see Table 1). As expected, female subjects’ adult dominance rank was not independent of their mothers’ dominance rank when the females were born ($\chi_{\left(4\right)}^{2}$ = 70.99, *p* < 0.001; see Table 2). More than half of the adult female subjects (58%) attained the same adult dominance rank that their mother held when the female subjects were born. As in females, males’ adult social dominance rank was not independent of their mother’s social dominance rank ($\chi_{\left(4\right)}^{2}$ = 20.60, *p* < 0.001; see Table 2), although the effect was not as strong as it was for females. For males, only high maternal dominance rank was statistically associated with the dominance rank the males attained as adults, with most high-ranking males (78%) born to mothers that also held high dominance rank at her son’s parturition. The trend, however, was similar to that seen in females, with more of the middle- and low-ranking adult males having middle- and low-ranking mothers at birth.

**Table 2:** Frequency counts and percent of subjects’ adult social dominance rank within maternal social dominance rank.

	Maternal dominance rank at birth			
Subjects’ adult rank	High-Ranking	Middle-Ranking	Low-Ranking	Marginal totals
Females
High-Ranking	51 (73%)*	**2 (6%)^†^**	**5 (12%)^†^**	58
Middle-Ranking	14 (20%)	19 (54%)*	13 (31%)	46
Low-Ranking	**5 (7%)^†^**	14 (40%)	24 (57%)*	43
Marginal totals	70	35	42	147
Males
High-Ranking	14 (78%)*	11 (31%)	10 (34%)	35
Middle-Ranking	**2 (11%)^†^**	23 (66%)	12 (41%)	37
Low-Ranking	2 (11%)	1 (3%)	7 (24%)	10
Marginal totals	18	35	29	82

The table shows cross-tabulated frequency counts of subjects’ maternal social dominance rank by the social dominance rank they achieved as adults. The percent of subjects’ adult dominance rank within maternal dominance rank is shown in parentheses. The bolded * indicates an observed count that is significantly greater than the cell’s expected count (standardized residual >2). The bolded † indicates an observed count that is significantly less than the cell’s expected count (standardized residual < −2).

### Analyses

Analyses were performed in SPSS, v.25 (IBM). To test for sex differences and hand differences (right-hand vs. left-hand) in 2D:4D ratio, repeated measures analysis of variance (RMANOVA) were performed with sex as a fixed factor, right-hand and left-hand as repeated measures, and 2D:4D ratio as the dependent variable. To examine the effect of maternal social dominance rank and subjects’ adult social dominance rank on 2D:4D ratio, RMANOVAs were performed with sex, subjects’ adult dominance rank, and maternal dominance rank as fixed factors, right-hand and left-hand as repeated measures, and 2D:4D ratio as the dependent variable. For analyses that yielded a significant interaction between subjects’ adult social dominance rank and sex or maternal dominance rank and sex, follow up simple-effect comparisons were performed to assess sex differences at each level of subjects’ adult dominance rank tertile or maternal dominance rank tertile. To compare the sex differences within each rank, RMANOVAs were performed for each of the three rank tertiles, with sex as a fixed factor, right-hand and left-hand as repeated measures, and a Bonferroni corrected alpha criterion of 0.016.

## Results

### Sex Differences and Hand Differences in 2D:4D Ratio

**Figure 1:**
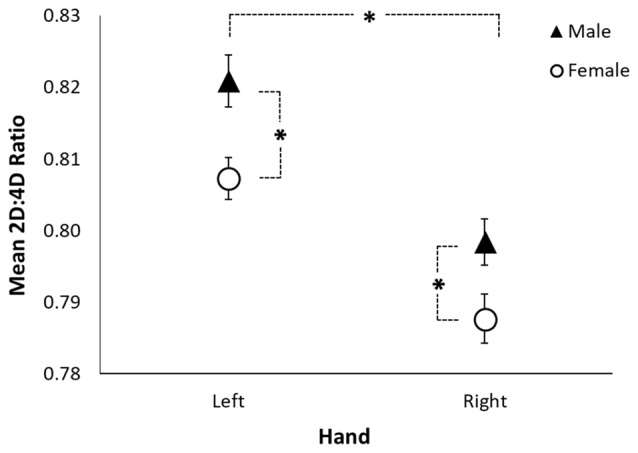
Sexual dimorphism in rhesus monkeys’ 2D:4D ratio. The figure shows the mean left-hand and right-hand 2D:4D ratio by sex. The black triangles represent the mean for males, the open circles represent the mean for females, the error bars represent ±1 standard error, and the asterisks represent a significant difference at *p* < 0.05.

A RMANOVA revealed a sex difference in 2D:4D ratio (*F*_(1,205)_ = 6.53, *p* = 0.011; see Table 3 and Figure 1) across both hands, with males exhibiting higher 2D:4D ratio than females. There was also a significant difference in 2D:4D ratio between hands (*F*_(1,205)_ = 35.37, *p* < 0.001): subjects’ left-hand 2D:4D ratio was higher than their right-hand 2D:4D ratio. There was no interaction between sex and hand.

**Table 3:** Results of RMANOVAs.

Variable	*DF* (between, within)	*F*	*p*
1. Two-way RMANOVA: hand by sex			
Hand*	(1, 205)	35.37	<0.001
Sex*	(1, 205)	6.52	0.011
Hand by sex	(1, 205)	0.01	0.93
2. Four-way RMANOVA: hand by sex by maternal rank by adult rank			
Hand*	(1, 131)	18.93	<0.001
Sex	(1, 131)	0.17	0.68
Maternal rank	(2, 131)	1.07	0.35
Adult rank	(2, 131)	0.24	0.79
Sex by maternal rank*	(2, 131)	4.10	0.019
Sex by adult rank	(2, 131)	1.69	0.19
Maternal rank by adult rank	(4, 131)	1.85	0.12
Sex by maternal rank by adult rank	(3, 131)	1.06	0.37
Hand by sex	(1, 131)	0.22	0.64
Hand by maternal rank	(2, 131)	0.69	0.50
Hand by adult rank	(2, 131)	0.04	0.96
Hand by sex by maternal rank	(2, 131)	0.28	0.75
Hand by sex by adult rank	(2, 131)	0.14	0.87
Hand by maternal rank by adult rank	(4, 131)	0.75	0.56
Hand by sex by maternal rank by adult rank	(3, 131)	0.30	0.82
3. Simple effects: ^a^two-way RMANOVA (offspring of low-ranking mothers)			
**Hand^†^**	(1, 41)	19.40	<0.001
**Sex^†^**	(1, 41)	9.125	0.004
Hand by sex	(1, 41)	0.18	0.673
4. Simple effects: ^a^two-way RMANOVA (offspring of middle-ranking mothers)			
**Hand^†^**	(1, 51)	10.034	0.003
Sex	(1, 51)	0.039	0.845
Hand by sex	(1, 51)	1.835	0.181
5. Simple effects: ^a^two-way RMANOVA (offspring of high-ranking mothers)			
**Hand^†^**	(1, 66)	9.33	0.003
Sex	(1, 66)	1.41	0.24
Hand by sex	(1, 66)	1.68	0.20

The table shows the results of the RMANOVAs used in analyses. *DF* indicates degrees of freedom (between-subjects, within-subjects); *F* indicates *F* ratio; *p* indicates significance. The bolded variable names marked with an * are significant at *p* < 0.05. The bolded variable names marked with a † are significant at *p* < 0.016 (Bonferroni correction for three comparisons). In all analyses, subjects’ 2D:4D ratio was the dependent variable, and right-hand and left-hand were included as repeated measures. In the first analysis (Two-Way RMANOVA: Hand by Sex), the independent variables used were sex, hand, and the interaction between sex and hand. In the second analysis (Four-Way RMANOVA: Hand by Sex by Maternal Rank by Adult Rank), maternal social dominance rank and subjects’ adult social dominance rank were added in the model, with all possible two-way, three-way and four-way interactions. ^a^Follow up analyses were performed to explore the interaction between sex and maternal dominance rank found in the Four-Way RMANOVA. In these simple effects analyses, the analysis described above for the first model (Two-way RMANOVA: hand by sex) was performed separately for each level of maternal dominance rank (low-ranking, middle-ranking and high-ranking). To correct for multiple comparisons, a Bonferroni corrected alpha criterion of *p* < 0.016 was used.

### Relationship Between Maternal Dominance Rank and 2D:4D Ratio

**Figure 2:**
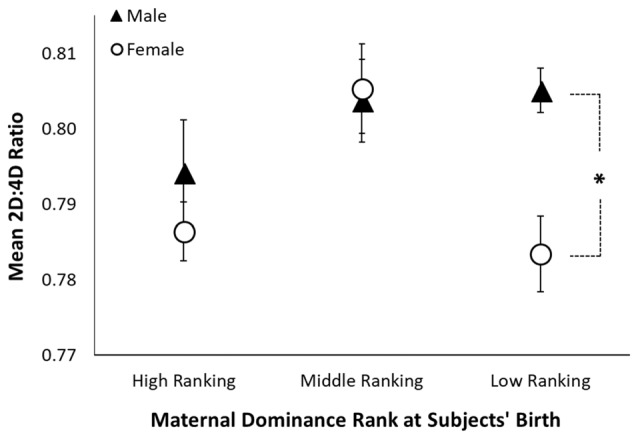
Sexual dimorphism in rhesus monkeys’ 2D:4D ratio is moderated by maternal dominance rank. The figure shows mean 2D:4D ratio (averaged across the right and left hands) by maternal dominance rank and sex. The black triangles represent the mean for males, the open circles represent the mean for females, the error bars represent ±1 standard error, and the asterisk represents a significant comparison at *p* < 0.05.

Across both hands, there was a significant interaction between sex and maternal dominance rank on 2D:4D ratio (*F*_(2,131)_ = 4.10, *p* = 0.019; see Table 3 and Figure 2). *A posteriori* planned comparisons revealed that, among offspring of low-ranking mothers, there was a significant sex difference in 2D:4D ratio across both hands (*F*_(1,41)_ = 9.13, *p* = 0.004, Bonferroni corrected alpha criterion = 0.016), such that females born to low-ranking mothers exhibited lower 2D:4D ratio than males born to low-ranking mothers. There was not a significant sex difference in 2D:4D ratio among offspring born to high-ranking or middle-ranking mothers (*p* > 0.19), nor were there any effects of subjects’ adult social dominance rank (see Table 3).

## Discussion

### Sexual Dimorphism in Rhesus Monkeys’ 2D:4D Ratio

As hypothesized, our data show a sex difference in rhesus monkeys’ 2D:4D ratio across both hands, with male rhesus monkeys exhibiting higher 2D:4D ratio than female rhesus monkeys. Our findings are consistent with other studies of Old World monkeys showing that males have a higher 2D:4D ratio than females (in rhesus monkeys: Abbott et al., 2012; in Guinea baboons: Roney et al., 2004). To our knowledge, this is the first report of a significant sex difference in rhesus monkeys’ 2D:4D ratio. Given the large sample size of males and females in this study, this finding suggests that PAE has an organizational effect on finger development in rhesus monkeys. Although other prenatal and postnatal factors may have contributed to this sex difference in 2D:4D ratio (for a thorough discussion of the benefits and limitations of 2D:4D ratio as a biomarker for PAE, see Breedlove, 2010), there is good evidence to support a PAE-based interpretation. For example, in one study, Abbott et al. (2012) found that, compared to control females, female rhesus monkeys that were administered high levels of prenatal testosterone during early gestation exhibited increased, male-typical 2D:4D ratio. Further, Abbott et al. (2012) found that the degree to which females’ 2D:4D ratio was increased (masculinized) was highly predictive of anogenital distance, an established biomarker of PAE (Thankamony et al., 2016). Although we did not directly measure prenatal hormones, and further research is needed to confirm our interpretation, bearing in mind our data and Abbott et al.’s (2012) findings, 2D:4D ratio is likely an index of PAE in rhesus monkeys, although its effects on 2D:4D ratio seem to be in the opposite direction compared to humans and apes, with high 2D:4D ratio indicative of high PAE in rhesus monkeys.

While somewhat speculative, the mechanism explaining the reversed pattern may be related to the distribution of androgen receptors in the fingers. As noted earlier, studies of rodents indicate that the human-like pattern of sex differences in 2D:4D ratio (males exhibiting lower 2D:4D ratio than females) may be explained by high concentrations of androgen receptors in the fourth digit and low concentrations in the second digit, leading high levels of PAE to selectively induce growth in the fourth digit without inducing growth in the second finger (Zheng and Cohn, 2011). However, as rhesus monkeys exhibit a reversed pattern of sexual dimorphism in 2D:4D ratio when compared to humans and mice, it is possible that concentrations of androgen receptors are reversed across digits in rhesus monkeys (see Lofeu et al., 2017). In support of this interpretation, in the aforementioned study, Abbott et al. (2012) found that experimentally administering prenatal androgens to female rhesus monkeys elongated and masculinized the second digit, leaving the fourth digit unaffected, and leading to a reversal of the 2D:4D ratio pattern. While a more direct investigation of androgen receptors is required to confirm this interpretation, this may explain why rhesus monkeys and some other Old World monkey species show a reversed pattern of sex differences in 2D:4D ratio when compared to humans and apes (see Lofeu et al., 2017). Further, Abbott et al. (2012) found that PAE increased 2D:4D ratio on the right hand (and not the left), which may indicate that right-hand 2D:4D ratio and left-hand 2D:4D ratio index slightly different prenatal periods of androgen exposure, or that androgen receptor concentrations differ between hands. This may explain, in part, why in our study, subjects’ left-hand 2D:4D ratio was not correlated with their right-hand 2D:4D ratio, and why, on average, their left-hand 2D:4D ratio was higher than their right-hand 2D:4D ratio. However, considering that lateral differences in 2D:4D ratio are not well-understood in humans (see Hönekopp and Watson, 2010) or nonhuman primates, and that studies of humans report a wide range of correlation coefficients between right-hand and left-hand 2D:4D ratio (see Hönekopp and Schuster, 2010), further research is needed to confirm this interpretation.

Regardless of the mechanism, from an evolutionary perspective, our findings add to a growing body of literature suggesting that the pattern of sexual dimorphism in 2D:4D ratio varies phylogenetically across primates. For example, in addition to our findings in rhesus monkeys, other monkey species like Guinea baboons show a similar pattern of reversed 2D:4D ratio (Roney et al., 2004), while great ape species, like chimpanzees and bonobos (McIntyre et al., 2009), exhibit human-like patterns of sex differences in 2D:4D ratio. It should be noted that some studies investigating sexual dimorphism in metacarpal length ratios among ape species have found that gorillas (*Gorilla gorilla gorilla*; see McFadden and Bracht, 2005) and gibbons (*Hylobates lar*; see Hart, 2018) do not exhibit sexual dimorphism in the second-to-fourth-metacarpal length ratio. However, as PAE may affect metacarpal growth and overall finger length differently, these two proxies for PAE may not be directly comparable (see McFadden and Bracht, 2005). Hence, based on finger-length ratio in the species studied thus far, patterns of 2D:4D ratio appear to be more consistent within primate families than between, which suggests that these family-level differences in 2D:4D ratio emerged when the common Old World monkey ancestor diverged from the common great ape ancestor. Further investigation across a wider variety of primate species is needed to confirm this interpretation, and may shed important light on the role PAE has played in the evolution of primate social systems (see Nelson and Shultz, 2010). We speculate that the reversed pattern of 2D:4D ratio in Old World monkeys compared to humans and great apes may have arisen from the same selective pressures on hand structure that also selected for other family-wide differences between Old World monkeys and hominoids, including traits like the high rates of tool use observed in hominoid species relative to Old World species (see van Schaik et al., 1999), or differences in arboreal- or terrestrial-living across primate species (see Milton and May, 1976). It should be noted that three studies of Old World species failed to find any sex differences in 2D:4D ratio. However, two of these studies, which investigated rhesus monkeys (Nelson and Voracek, 2010; Abbott et al., 2012), may have been underpowered to detect sex differences in 2D:4D ratio (see footnote 1). The third study did not find a significant sex difference in 2D:D ratio, despite investigating a large sample^3^ of Hamadryas baboons (Huber et al., 2017). However, in that study, the direction of the means was in the expected direction for Old World monkeys, with males exhibiting higher 2D:4D ratio than females (Huber et al., 2017). These null findings suggest that the association between PAE and 2D:4D ratio is complex, and there may be moderating factors. Regardless of the underlying ultimate mechanism, our data show that in comparison to humans and great apes, rhesus monkeys show a reversed pattern of sexual dimorphism in 2D:4D ratio, a pattern which may be characteristic of other Old World monkeys. Further research is needed in other species using large sample sizes to determine whether this is a general pattern seen across other monkey species.

### Association Between 2D:4D Ratio and Maternal Dominance Rank

To our knowledge, this is the first study to investigate 2D:4D ratio and social dominance rank in male rhesus monkeys, as well as the first to investigate the relationship between maternal dominance rank and offspring 2D:4D ratio in a large sample size of rhesus monkeys. As predicted, 2D:4D ratio varied with maternal dominance rank. We found that among offspring born to high-ranking and middle-ranking mothers, females’ 2D:4D ratio was generally higher (more male-typical) than that exhibited by females born to low-ranking mothers, and did not differ when compared to males born to mothers of the same respective rank. However, among offspring of low-ranking mothers, females exhibited lower (more female-typical) 2D:4D ratio than males, a pattern reflected in the larger sample. Although speculative, to the extent that 2D:4D ratio reflects PAE in rhesus monkeys (see Abbott et al., 2012), this finding is consistent with a maternal dominance rank-based androgen effect (see Nelson et al., 2012). Some studies suggest that high-ranking and middle-ranking mothers have higher levels of circulating testosterone than low-ranking mothers (Rose et al., 1971; Sapolsky, 1983), which may masculinize their offspring’s 2D:4D ratio throughout pregnancy. Although maternal rank-based androgen effects would theoretically have similar effects on male and female offspring, we found no effect of maternal dominance rank on male offspring’s 2D:4D ratio, suggesting a ceiling effect in males, such that the high levels of prenatal androgens produced by males’ testes may have obscured any greater effects of maternal rank-based androgen exposure. While speculative, it is possible that dominance rank-based alterations in maternal hormones during pregnancy contribute, in part, to the dominance rank that female rhesus monkeys ultimately attain as adults (Nelson et al., 2012).

## Conclusion

Using a large sample of rhesus monkeys, our data show a sexually-dimorphic 2D:4D ratio pattern that is consistent with other studies of Old World monkeys (Roney et al., 2004; Abbott et al., 2012), with males exhibiting higher 2D:4D ratio than females. This pattern is reversed when compared to humans and great apes, and based on the species studied thus far, may characterize 2D:4D ratio in Old World monkeys. A primary means of addressing the underlying monkey vs. hominid difference in 2D:4D ratio and its biological basis would be to measure androgen receptor density in the index and ring fingers in humans and across a variety of Old World nonhuman primate species. Continued investigation of 2D:4D ratio in rhesus monkeys may lead to a better understanding of the evolution of sexually-dimorphic central nervous system traits in humans and across primate phylogeny. To the extent that 2D:4D ratio correlates with PAE in rhesus monkeys (see Abbott et al., 2012), future research using this biomarker may shed light on the role that PAE plays in psychiatric disorders (see Kalin and Shelton, 2003; Capitanio, 2017; Parker et al., 2018).

Our results also show an association between 2D:4D ratio and maternal social dominance rank. This finding suggests that maternal dominance rank contributes to the level of PAE experienced by offspring. Nonhuman primates will likely provide a useful model for continued investigation using more controlled procedures and direct measure of prenatal hormones than is currently possible in humans. Moreover, as many variables contribute to social dominance rank, one interesting line of research would be to study whether PAE affects specific temperament traits, which may explain, in part, the variation in social dominance rank seen in rhesus monkeys and in other species, as well as the effects of social dominance rank on maternal androgen levels in rhesus monkeys.

## Data Availability

The raw data supporting the conclusions of this manuscript will be made available by the authors, without undue reservation, to any qualified researcher.

## Author Contributions

AB, EW, AC, JC and JH formulated the study design and methodology. AB and EW collected finger measurement data. AC collected social dominance rank data. AB, EW and JH analyzed the data. AB, EW and JH wrote the first draft of the article. PJ assisted with subsequent drafts. All authors contributed to revising the final draft.

## Conflict of Interest Statement

The authors declare that the research was conducted in the absence of any commercial or financial relationships that could be construed as a potential conflict of interest.

## Footnotes

^1^Abbott et al. (2012) compared 19 untreated laboratory-living adult females to nine adult males; Nelson and Voracek (2010) compared eight untreated free-ranging infant females to 17 infant males.

^2^Nelson and Shultz (2010) investigated 242 female rhesus monkeys and 53 male rhesus monkeys from five institutions.

^3^Huber et al. (2017) compared 33 males to 77 females.

## Abbreviations

CNPRC: California National Primate Research Center
PAE: Prenatal androgen exposure
2D:4D ratio: second-to-fourth-finger-length ratio
RMANOVA: repeated measures analysis of variance.
